# The Diversity in Grapes of *Vitis labrusca* Grown in Bolu (Türkiye) Assessed by Multivariate Approaches

**DOI:** 10.3390/genes14071491

**Published:** 2023-07-21

**Authors:** Emrah Güler, Emre Kan, Mehmet Settar Ünal

**Affiliations:** 1 Department of Horticulture, Faculty of Agriculture, Bolu Abant Izzet Baysal University, Bolu 14030, Türkiye; 2 Hazelnut Specialization Coordinatorship, Giresun University, Giresun 28200, Türkiye; emre.kan@giresun.edu.tr; 3 Institute of Graduate Studies, Bolu Abant Izzet Baysal University, Bolu 14030, Türkiye; 4 Department of Horticulture, Faculty of Agriculture, Sirnak University, Sirnak 73000, Türkiye; munal62@hotmail.com

**Keywords:** total phenolic content, morphometrics, must properties, antioxidants, confirmatory factor analysis, PCA, HCA

## Abstract

The grape is one of the most produced and processed horticultural crops. This study evaluated the grape genetic resource belonging to the *Vitis labrusca* species. The diversity was assessed according to morphometric, antioxidant, physicochemical, and colorimetric characteristics. The diversity was evaluated using a variation index and multivariate analyses. The bunch weight of the vines exhibited a range from 21.05 g to 162.46 g, with a coefficient of variation (CV) of 38.97%. The average bunch weight was 64.74 g. In terms of the berry properties, the highest CV was observed for the berry weight (21.95%). The peel thickness displayed a CV of 36.40%, and an average of 0.23 mm. The CVs for the juice characteristics in the berries of the studied vines were 7.11%, 16.61%, 19.41%, and 28.10% for the pH, TSS, must yield, and TA, respectively. The TPC of the accessions exhibited a notably low variation (CV = 4.63%). The color properties of the accessions displayed an immense variation, except for the L* values. The hierarchical clustering analysis divided the accessions into two main clusters, which both had two subclusters. The multivariate approaches separated individuals into different groups, and they were considered useful tools for utilization in the genetic diversity assessments. Further studies on the cultivation technique and crossbreeding with *Vitis vinifera* will provide more insights into the population, and this study will be a source for upcoming studies on *V. labrusca* in the region.

## 1. Introduction

Due to its favorable climatic conditions, Türkiye is recognized as one of the gene centers for *Vitis vinifera* grapes worldwide. The country possesses valuable grape gene resources, with more than 1500 grape varieties cultivated in the National Grape Gene Resources Centers [1,2,3]. In terms of grape production, Türkiye ranks sixth globally, and fifth in terms of vineyard area, with 390,221 hectares of vineyards, and an annual grape production of 3,670,000 tons [4]. Alongside *V*. *vinifera* varieties, Türkiye also boasts a diverse genotype of *V. labrusca* L. species. *V. labrusca* L. grapes are predominantly grown in the central and eastern Black Sea Region, situated in the northern part of the country [5,6,7]. These grapes are characterized by a distinct aroma, a foxy flavor, a thick slip skin, and jelly-like flesh. Due to their high market value, *V. labrusca* L. grapes are widely sold as table grapes in local markets in the cities along the coast of the Black Sea [8]. They are consumed by local residents as table grapes, marmalades, grape molasses, pickles, jams, or juices, depending on local preferences [9,10,11,12,13]. Numerous studies have demonstrated the resistance of *V. labrusca* L. grapes to fungal diseases such as powdery mildew and downy mildew [12,14,15,16]. The vines of *V. labrusca* grow by climbing around trees, in home gardens, as a canopy or pergola, or extending to the balconies of buildings and houses.

The examination of the various characteristics that demonstrate their range of diversification holds significant importance in terms of agronomy and food production [17]. Through the observation of variations within a population, superior types can be selected, based on their resistance to biotic and abiotic stressors, suitability for cultivation, and ability to yield high-quality crops [18]. Phenotyping methods, which involve assessing the shoot and leaf traits, bunch characteristics, berry color and shape, and chemical composition, are commonly employed to characterize the grape germplasm, and distinguish between different varieties [19]. Morphological features are particularly sensitive to environmental changes, and ongoing climate change, characterized by rising temperatures, floods, and droughts, further underscores the need for morphological characterization studies [20]. Therefore, the determination of stable characteristics that are relatively less affected by environmental change is a necessary element to utilize in breeding studies, during the selection process [21].

*V. labrusca* L. grown in Türkiye are known as Isabella. However, this species has spread to all the Black Sea coasts of Türkiye, and many types have emerged. Recently, five different varieties have been introduced in Samsun, the central Black Sea region [22]. Both the increase in the demand for *Labrusca* grapes on the market, and the fact that diseases such as gray mold and powdery mildew prevent *V. vinifera* vines from growing in the region have increased the interest in this species [23]. In this regard, this study was conducted to perform a preliminary diagnosis of the morphometric, antioxidant, physicochemical, and colorimetric characteristics of the *V. labrusca* L. population grown in Bolu, a province in the western Black Sea region of Türkiye.

## 2. Materials and Methods

### 2.1. Plant Material and Research Site

The vines of *V. labrusca* grown in Bolu (Türkiye) comprised the materials for this study. The vines of this species are known as Isabella in the region. A total of 72 accessions were determined by a survey, and labeled from 1 to 72 (I-1 to I-72) in July. Bunches from the labeled vines were gathered in early September, and were brought directly to the laboratories of the Department of Horticulture, at the Faculty of Agriculture, Bolu Abant İzzet Baysal University.

Bolu province is located in the humid region. Regarding long-term climate data, the annual precipitation varies between 500–1500 mm. The rainfall is highest in January, May, and December, and lowest in July and August. The average number of snowy days is 38.9. August is the hottest month, with an average of 20 °C. The soil in the region is primarily loamy, having a high proportion of sand. The pH varies from slightly acidic to slightly alkaline. The organic matter content in the upper layers varies from moderate to sufficient [24].

### 2.2. Measurement of Morphometric Characteristics and Physicochemicals

The bunch characteristics were determined by measuring five bunches from each vine. The length, width, and stalk length of the bunches were measured using a digital caliper, and expressed in millimeters (mm). The bunch weight was measured using a digital scale, and expressed in grams (g). The berry characteristics were defined through the measurement of 50 berries from each vine. The berry weights were assessed using a digital scale, and the results were recorded in g. The berry width, length, and peel thickness were measured using a digital caliper, and expressed as mm. The number of seeds in each berry was counted and recorded. The must yield was calculated through the extraction of juice from the berries, and the division of the resulting must by the total weight of the berries, then multiplication by 100 to obtain the percentage.

For the physicochemical analyses, 10 g samples were taken from each replication, and their seeds were removed. The samples were then blended using an electric blender. The total soluble solids (TSSs) were determined using a digital refractometer (ATC, 0–32, Turkey), and the values were expressed as percentages. For the titratable acidity, a sufficient volume of juice was taken, and titrated with 0.1 N NaOH, with the results reported as the grams of tartaric acid per liter [25]. The pH of the juice was measured using a pH meter (Orion Star A211, Thermo Scientific, Waltham, MA, USA).

### 2.3. Determination of Total Phenolic and DPPH Scavenging Activity

To determine the total phenolic content, the microscale procedure reported by Waterhouse [26] was used, with modifications. Briefly, 1600 µL of distilled water and 50 µL of Folin–Ciocâlteu agent were added to 50 µL of methanolic extract and mixed gently. Then, 300 µL of 7% (*w*/*v*) calcium carbonate solution was added and vortexed. After the mixture was left in the dark at room conditions for 2 h, its absorbance at 760 nm was read, using a UV–Vis spectrophotometer (SP-UV1100, DLAB, Beijing, China). The obtained absorbance values were converted to real content through the calculation of the equation obtained with the standard curve (R^2^ = 0.99) prepared using 0.5, 1, 2, 3, 4, 5, and 6 mM gallic acid with the same procedure.

The 2,2 Diphenyl 1 picrylhydrazyl obtained from Sigma-Aldrich (Darmstadt, Germany) was prepared in ethanol, with a final absorbance within the range of 0.7–0.8, to measure the DPPH scavenging activity. Then, the activity was measured using the following procedure. The most appropriate methanolic extract amount was determined through preliminary trials, with a final volume of 2 mL; 50 µL sample, 1450 µL ethanol, and 500 µL of DPPH solution were added sequentially and vortexed. The prepared solution was measured at a 520 nm wavelength using a UV–Vis spectrophotometer after 15 min, and the DPPH scavenging capacity was calculated using the following formula.

DPPH (%) = (A_blank_ − As_ample_)/A_blank_.

### 2.4. Total Monomeric Anthocyanin Measurement

The pH differential method defined by Fuleki and Francis [27] was applied, with some modifications, for the determination of the total monomeric anthocyanin. Briefly, the appropriate amount to be taken from the methanolic extract was determined, and the dilution factor was recorded, to obtain an absorbance in the range of 0.4–0.8 at the maximum wavelength. Then, 0.4 mL of prepared dilution was decanted into two separate tubes, and these were filled to 2 mL with pH 1.0 and pH 4.5 buffer solutions. The tubes were then capped, and kept at +4 °C for 2 h in dark conditions. The samples were measured at wavelengths of 516 nm and 700 nm, and the true absorbance was calculated using the following formula:

Absorbance (A) = (A_516_-A_700_) pH 1.0 − (A_516_-A_700_) pH 4.5.

The total amount of monomeric anthocyanin was calculated by adding the obtained absorbance value to the formula below.

Total anthocyanin (mg/L) = (A × 10^3^ × MW × DF)/(E × L)

A: absorbance,
MW: molecular weight of pigments, (cyanidin 3 glucoside; 484,83 g/mol),
DF: dilution factor,
E: molar absorbance (26,900),
L: the optical path (1 cm) of the cuvette.

### 2.5. Color Properties

The numerical color values in the CIE color space—the L*, a*, b*, Chroma, and Hue angle values—were determined using a handheld colorimeter (PCE, UK). The anthocyanin degradation method was used to determine the color intensity and polymeric color [28]. During the sample preparation, the dilution, and absorbance values were adjusted as in total monomeric anthocyanin, and a 0.4 mL sample was placed in two different tubes; one was filled with distilled water, and the other with 20% (*w*/*v*) metabisulfite solution. Both solutions were measured half an hour later, using a UV–Vis spectrophotometer at 420 nm, 516 nm, and 700 nm. The color density was calculated in the untreated sample, and the polymeric color was computed in the bisulfite-treated sample, using the following formula.

Color density/polymeric color = [(A_516_ − A_700_) + (A_420_ − A_700_)] (DF).

DF: dilution factor

### 2.6. Statistical Evaluations

Each sample was analyzed as three technical replicates. The data were subjected to Levene’s homogeneity test to ensure that they were homogeneous. After the normality was determined, the data were subjected to Tukey’s post hoc test (HSD), with an α level of 0.05. The correlations across traits were determined using the “corrplot” package of R Studio according to Pearson’s pairwise correlations. The interrelations among individuals and the studied traits were evaluated through principal component (PCA) and heatmap analyses, using the “ggplot2” package of R Studio [29]. The dependencies among the morphometrics, color properties, antioxidants, and must properties were evaluated through path analysis, using CFA (confirmatory factor analysis) in the “Lavaan” package of the R Studio software [30].

## 3. Results

### 3.1. Morphological Diversity

The bunch weight of the vines under study exhibited a range from 21.05 g to 162.46 g, with a coefficient of variation (CV) of 38.97%. The average bunch weight was 64.74 g. The bunch stalk length values varied between 35.80 mm and 65.00 mm, with a CV of 41.00%, and an average length of 29.98 mm. The average bunch length was measured as 140.80 mm, with a minimum of 41.00 mm and a maximum of 235.00 mm. The CV for the bunch length was calculated as 17.26%. The bunch width ranged from 30.00 mm to 94.00 mm, with an average CV of 23.75%, and a mean width of 46.74 mm (Table 1).

In terms of the berry properties, the highest CV was observed for berry weight (21.95%). The average berry weights ranged from 1.01 g to 3.03 g, with a mean of 1.89 g. The berry lengths exhibited a range from 10.00 mm to 18.27 mm, with an average length of 15.41 mm. The CV for berry length was calculated as 9.61%. The mean berry width was 13.73 mm, with a CV of 7.38%. The minimum and maximum values for berry width were 10.71 mm and 16.27 mm, respectively. The peel thickness displayed a CV of 36.40%, and an average of 0.23 mm. The minimum and maximum peel thickness values were 0.10 mm and 0.69 mm, respectively. The number of seeds per berry ranged from 1.00 to 3.00, with an average of 1.73, and a CV of 31.25% (Table 1).

### 3.2. Berry Physicochemical Characteristics

The CVs for juice characteristics in the berries of the studied vines were 7.11/, 16.61%, 19.41%, and 28.10% for the pH, TSS, must yield, and TA, respectively. The TSS was in the range of 10.00–21.20%, with a mean of 16.61%. The mean pH was 3.33, and ranged from 2.94 to 4.65. The TA varied between 0.32 mg/L and 1.94 mg/L, with a 0.76 mg/L mean value. The juice yield in the berries of the studied vines of *V. labrusca* varied from 28.83% to 81.05%, with a mean of 48.74% (Table 1).

### 3.3. Antioxidant Properties

The TPC of the accessions exhibited a notably low variation (CV = 4.63%). The mean TPC was calculated as 31.75 mg/g, and ranged from 24.92 mg/g to 34.64 mg/kg. The total antioxidant capacity determined using the DPHH scavenging activity showed a relatively high variation (CV = 25.82%), and varied between 25.20% and 80.30%, with a mean of 55.47%. The total anthocyanin content, assessed using the pH differential method, displayed the highest CV (36.81%) among the antioxidant properties of *V. labrusca* berries. The mean anthocyanin content was 37.78 mg/L, and the minimum and maximum contents were 16.37 mg/L and 83.50 mg/L, respectively (Table 1).

### 3.4. Colorimetric Traits

The color properties of the accessions displayed an immense variation, except for the L* values, which had a CV of 9.09%. The L* value ranged from 9.56 to 25.95, with a mean of 20.53. The mean values of a*, b*, Chroma*, and Hue^o^ were 0.45, −0.93, 2.52, and 250.73, respectively, while the CVs were 171.02%, 87.19%, 644.91%, and 34.20%. The color density of the juice of the berries ranged from 0.02 to 1.05, with a mean of 0.59, and a CV of 44.43%. The polymeric color had a mean of 0.86 with a CV of 32.44%, whereas the polymeric color ratio exhibited a 75.08% mean, and a 55.64% CV (Table 1).

### 3.5. Correlations among Studied Characteristics

**Figure 1:**
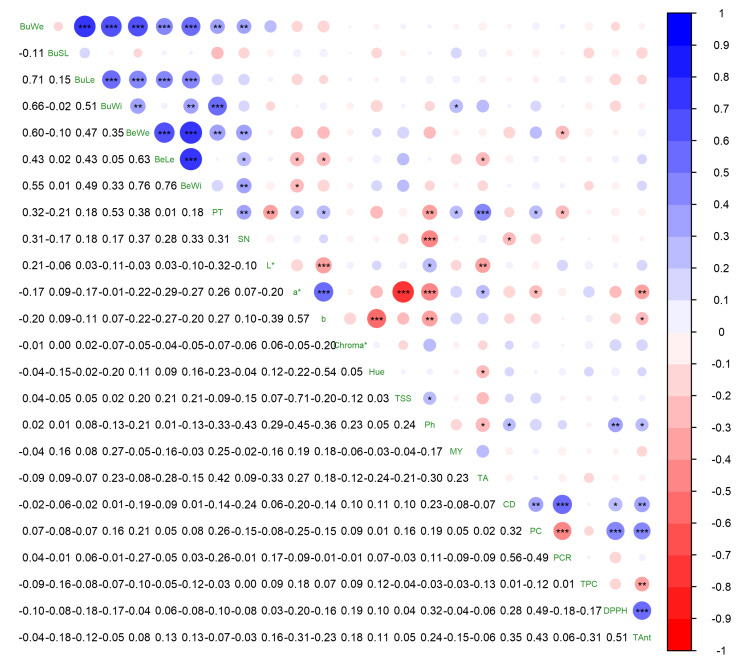
Correlations among the studied characteristics in berries of *V. labrusca* grown in Bolu. *, **, and *** indicates significance at *p* ≤ 0.05, *p* ≤ 0.01, and *p* ≤ 0.001, respectively. BuWe: bunch weight; BuSL: bunch stalk length; BuLe: bunch length; BuWi: bunch width; BeWe: berry weight; BeWi: berry width; BeLe: berry length; PT: peel thickness; SN: seed number per berry; TSS: total soluble solids; MY: must yield; TA: titration acidity; CD: color density; PC: polymeric color; PCR; polymeric color ratio; TPC: total phenolic content; TAnt: total anthocyanin content.

The berry morphometrics displayed positive correlations, except for the bunch stalk length. The size and weight characteristics exhibited strong correlations among themselves, generally greater than r = 0.70. The seed number was positively correlated to the size and weight properties. However, even though some correlations were significant, they were moderate to weak. The a* and b* values showed negative but negligible correlations with size and with parameters. These color traits demonstrated a relatively high positive correlation within themselves. The pH showed significant negative correlations with a*, b*, seed number, and peel thickness, while exhibiting positive correlations with the DPPH scavenging activity and total anthocyanin content. The color density was positively correlated with the polymeric color, polymeric color ratio, DPPH scavenging activity, and anthocyanin content. Interestingly, the total phenolic content only showed a significant correlation, which was negative, with the total anthocyanin (Figure 1).

### 3.6. Principal Component Analysis

**Figure 2:**
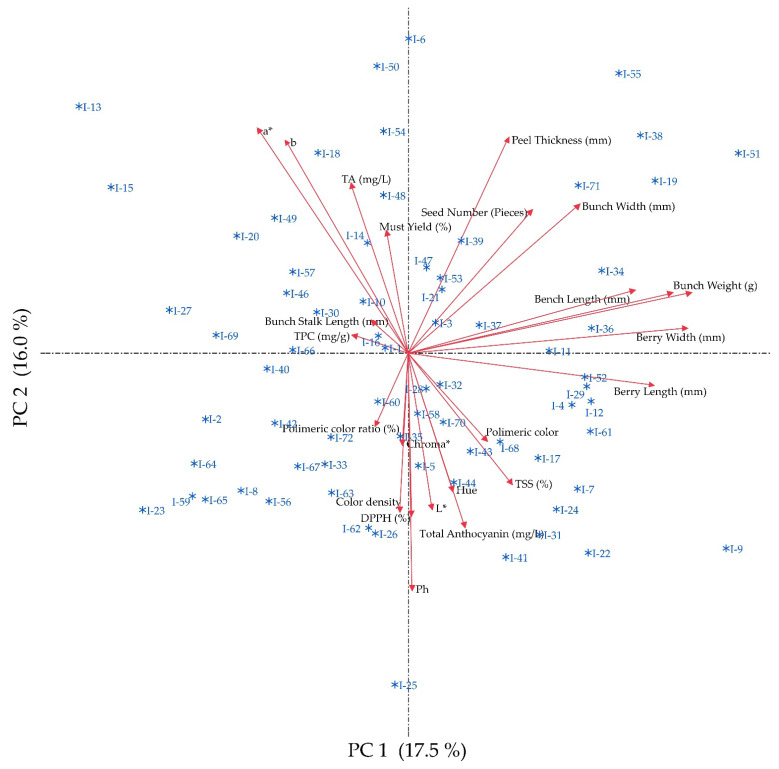
The biplot PCA analysis that illustrates the distribution of the accessions, according to the first two components. TA: titration acidity; TPC: total phenolic content.

The first ten principal components explained a cumulative variance of 80.41%, with the first three contributing 17.48%, 16.00%, and 9.69% to the total variance, respectively. Notably, 23 of the 24 principal components passed the Bartlett test, indicating their significance in explaining the variation observed. The first principal component was primarily influenced by the morphometric properties of the bunches and berries, while the second component was characterized by the negative contributions from the pH, DPPH scavenging activity, L* value, and color density, as well as the positive contributions from a*, b*, peel thickness, and TA. The accessions were widely spread in the biplot PCA analysis. The I-9 was distinct, with high vector loadings of morphometrics, physicochemical, and antioxidant properties, while the I-25 was separated by high loadings of physicochemical and antioxidant properties only (Figure 2).

### 3.7. Clustering and Heatmap Analysis

**Figure 3:**
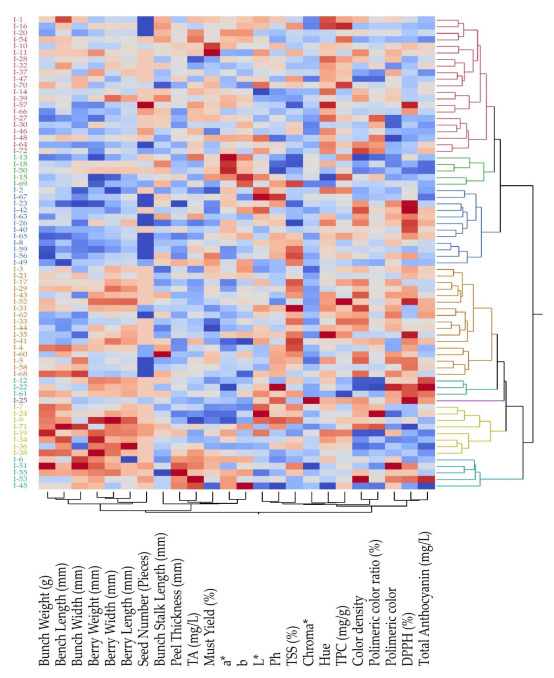
The clustering of the traits and the vines of *Vitis labrusca*. TA: titration acidity; TSS: total soluble solids; TPC: total phenolic content.

The hierarchical clustering analysis divided the accessions into two main clusters, which each had two subclusters. The studied genotypes were divided into eight different subclusters in total. The I-25 genotype formed an individual subcluster, while the I-12, I-22, and I-61 formed a subcluster characterized by moderate morphometric values, and high levels of polymeric color, DPPH scavenging activity, and total anthocyanin content. The seventh subcluster predominantly exhibited high values of morphometrics, while relatively low values were observed for antioxidant properties. Additionally, the morphometrics were essentially distinct from other characteristics when the accession were clustered (Figure 3).

### 3.8. Confirmatory Factor Analysis

**Figure 4:**
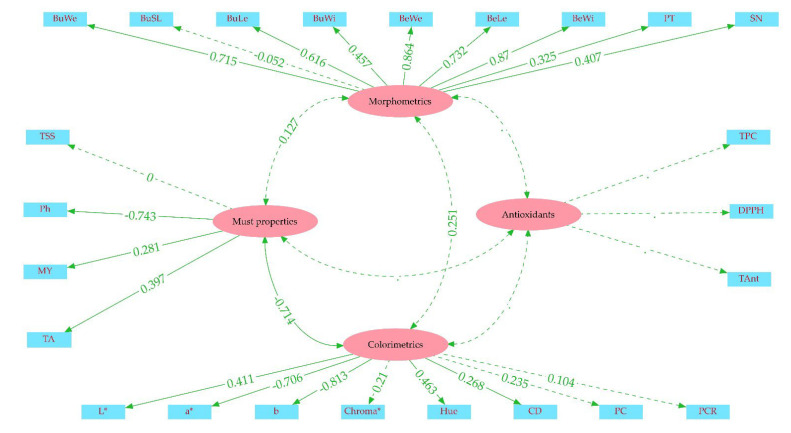
Path diagram for the confirmatory factor analysis. The light blue rectangles are traits, and the purple ellipses are latent groups. The straight green lines are loadings, and the curved lines are covariances. BuWe: bunch weight; BuSL: bunch stalk length; BuLe: bunch length; BuWi: bunch width; BeWe: berry weight; BeWi: berry width; BeLe: berry length; PT: peel thickness; SN: seed number per berry; TSS: total soluble solids; MY: must yield; TA: titration acidity; CD: color density; PC: polymeric color; PCR; polymeric color ratio; TPC: total phenolic content; TAnt: total anthocyanin content.

For the CFA, we divided the berry characteristics into four main latents: the morphometrics, must properties, antioxidants, and colorimetrics. The morphometrics included the bunch and berry size traits, bunch and berry weights, peel thickness, and seed number. Among the morphometrics, only the bunch stalk length displayed an insignificant loading on the latent, with a value of −0.052, while the berry width and weight exhibited the highest loadings, which were 0.870 and 0.864, respectively. The TSS was the only insignificant trait affecting the must properties latent, with a zero loading. The pH showed the highest effect, but it was negative (−0.743), while the must yield (0.281) and TA (0.397) positively contributed. The latent created for antioxidant properties did not include any significant traits. On the other hand, all the instrumental color values displayed significant loadings on the colorimetrics latent, whereas only color density showed a significant relationship across the spectrophotometric color features. The highest contribution was provided by the b* value (−0.813), followed by the a* value (0.706). Apart from these two negative factors, the others were positively related to the colorimetric latent group. There was only one significant covariance across the four latent groups, which was between the must properties and colorimetrics (*cov* = −0.714). The other relationships were not higher than *cov* = 0.251 (Figure 4).

## 4. Discussion

Berry sizes are influenced by a variety of factors, including the seed presence, pollination, and environmental circumstances [31]. The berry size in wild vines is known to fluctuate significantly during cultivation. The berry diameter in wild vines does not exceed 8 mm; however, the berry widths of farmed vines can range from 8 to 40 mm [32]. The berry widths in the *V. labrusca* accessions in this study ranged from a minimum of 10.00 mm to a maximum of 16.27 mm, which suggests that these vines were not wild types. The berry weight of a *V. labrusca* grape, Concord, was reported as 2.52 g by Cawthon and Morris [33]. Another study on *V. labrusca* reported a berry weight of 3.0 g and a bunch weight of 100 g in the Isabel variety in Brazil [34]. Previous research on *V. labrusca* reported higher values than we obtained in this study. The main reasons for such differences may be climatic factors and the genetic material. Brazil is one of the homelands of *V. labrusca* grapes, and there may have been artificial selection conducted by native peoples, resulting in higher quality grapes. The berry size is another characteristic that influences grape variety consumption. Because customers like large berries, genotypes with large berries are thought to be better suited for fresh eating. The berry size and weight characteristics were found to be significantly correlated, and this attribute will not be overlooked when we determine which approach to our genotypes will be best for future application.

The presence of seeds is an important component in determining the morphology of the fruit, and altering its biochemical content [35]. The hormones synthesized in the seed (auxins, gibberellins, brassinosteroids, cytokinins, polyamines, ethylene, and others) regulate seed development, and increase the activity and strength of the fruit, by acting as a co-factor determining the fruit size [36,37]. Furthermore, the seed properties (width, height, form index, volume, and so on) are important variables that are widely employed in ampelography investigations to identify genetic resources [38]. A slight variation in the seed existence indicates a high heredity rate of seed number in grapes, as demonstrated by Dolkar et al. [39], making it an important distinguishing feature in selection research.

One of the most important aspects when distinguishing grape types is the heterogeneous ripening behavior. Not all biological processes occur simultaneously in plant organs, and berries within the same cluster may not mature uniformly [40]. Some of the circumstances that can produce uneven maturation are the location of the vine in the vineyard, and the position of the cluster on the vine [41]. While consumers favor high TSS levels in cherries, kiwi fruit, peaches, and some grape varieties [42,43], the maturity is not only determined by the TSS levels. Solely considering the TSS is not an appropriate method of evaluating grapes, which have a fairly varied range of applications. The harvest level for excellent wines in cooler regions (the short season) is generally recommended to be between 18 and 22% [44,45]. Given the short vegetative season in the Bolu province, five of the genotypes investigated are appropriate for winemaking, in terms of the dry matter content (Table S1).

The total phenolic content of *V. labrusca* (var. Isabella) was reported as being considerably higher than that of the *V. vinifera* species by Rockenbach et al. [46]. A phenolic content ranging from 65 mg/100 g FW to 390 mg/100 g FW was reported by Abe et al. [47] in five Brazilian grapes belonging to *V. vinifera* and *V. labrusca*. The researchers also stated that the darker berries represented elevated values of anthocyanins, total phenolics, and antioxidant capacity. In this study, we also found that the color density and polymeric color were positively correlated to the total anthocyanin content. However, the total phenolic content and antioxidant scavenging capacity were not significantly affected by the color through the colorimetric properties; this was probably due to the high CVs of the color properties.

## 5. Conclusions

We identified a *V. labrusca* genetic source in the Bolu province of Türkiye. This is the first study that has defined the diversity in *V. labrusca* in the western Black Sea Region of Türkiye. The results indicated an enormous diversity in the color parameters across individuals. Contrarily, the morphometrics were relatively stable. We also presented a broad multivariate approach to the genetic diversity assessment of *V. labrusca* for the first time. The multivariate approach separated individuals into different groups, and was considered a useful tool to be utilized in genetic diversity assessment. This study will be a source for upcoming studies on *V. labrusca* in the region, and a comprehensive guide in terms of using a multivariate approach.

## Figures and Tables

**Table 1:** Descriptive statistics of *V. labrusca* accessions grown in Bolu.

Characteristics	Mean	Std Dev	Min	Max	CV (%)
Bunch weight (g)	64.74	25.23	21.05	162.46	38.97
Bunch stalk length (mm)	29.98	10.73	3.00	65.00	35.80
Bench length (mm)	140.80	24.30	41.00	235.00	17.26
Bunch width (mm)	46.74	11.10	30.00	94.00	23.75
Berry weight (mm)	1.89	0.41	1.01	3.03	21.95
Berry length (mm)	15.41	1.48	10.00	18.27	9.61
Berry width (mm)	13.73	1.01	10.71	16.27	7.38
Peel thickness (mm)	0.23	0.08	0.10	0.69	36.40
Seed number (pieces)	1.73	0.54	1.00	3.00	31.25
TSS (%)	14.95	2.48	10.00	21.20	16.61
Ph	3.33	0.24	2.94	4.65	7.11
TA (mg/L)	0.76	0.21	0.32	1.94	28.10
Must yield (%)	48.74	9.46	28.83	81.05	19.41
Total phenolic content (mg/g)	31.75	1.47	24.92	34.64	4.63
DPPH scavenging activity (%)	55.47	14.32	25.20	80.30	25.82
Total anthocyanin content (mg/L)	37.78	13.72	16.37	83.50	36.31
L*	20.53	1.87	9.56	25.95	9.09
a*	0.45	0.78	−1.17	3.28	171.02
b	−0.93	0.81	−2.76	1.42	87.19
Chroma*	2.52	16.23	−0.64	243.00	644.91
Hue	250.73	85.75	0.13	358.42	34.20
Color density	0.59	0.26	0.02	1.05	44.43
Polymeric color	0.86	0.28	0.27	1.51	32.44
Polymeric color ratio (%)	75.08	41.77	1.54	222.67	55.64

## Data Availability

The corresponding author can provide the data upon reasonable request.

## Supplementary Materials

The following supporting information can be downloaded at: https://www.mdpi.com/article/10.3390/genes14071491/s1, Table S1: Mean values of the genotypes in terms of studied characteristics.
